# Central Aspects of Tinnitus: Advances in Mechanisms and Neuromodulation

**DOI:** 10.3390/brainsci14090889

**Published:** 2024-08-31

**Authors:** Jasper V. Smit, Marcus L. F. Janssen

**Affiliations:** 1 Department of Ear Nose and Throat, Head and Neck Surgery, Zuyderland Medical Center, 6419 PC Heerlen, The Netherlands; 2 Department of Clinical Neurophysiology, Maastricht University Medical Center, 6229 HX Maastricht, The Netherlands; m.janssen@maastrichtuniversity.nl; 3 Mental Health and Neuroscience Research Institute, Maastricht University, 6229 ER Maastricht, The Netherlands

Tinnitus is a complex neuro-otologic disorder with a significant global impact, affecting approximately 14% of adults, with 2% experiencing severe forms [1]. Significant advancements have been made in understanding tinnitus. The occurrence of tinnitus is often linked to peripheral damage due to age-related hearing loss, exposure to loud noise, or certain pharmacological agents. For many years, tinnitus was believed to be solely a result of this peripheral damage, with the inner ear considered the neural generator for the condition. Current evidence shows altered neural activity throughout the central auditory pathway, including the dorsal cochlear nucleus (DCN), thalamus, and even non-auditory structures like the limbic system.

This Special Issue presents six papers that delve into the central mechanisms of tinnitus and explore neuromodulative treatments. 

Safazadeh and colleagues investigated sound-evoked cortical activity in persons with tinnitus and clinically normal hearing, focusing on potential differences in the tonotopic organization of the auditory cortex using MRI (contribution 1). The sound-evoked response in the hyperactivated cortical regions was positively correlated with tinnitus burden and hyperacusis scores, particularly for low frequencies. No differences in tonotopic organization were found, suggesting that hearing loss, rather than tinnitus, drives changes in the auditory cortex. This study thereby emphasizes the complex neural alterations in persons with tinnitus and the frequently observed comorbid hearing loss and hyperacusis. 

Attention may act as a gate, controlling the conscious awareness and processing of tinnitus. Richardson and colleagues hypothesized that the difference in cortical responses between attended and unattended conditions would be greater in tinnitus subjects (contribution 2). In the passive listening condition, there were no differences in cortical responses between tinnitus and control subjects. However, in the attentive listening conditions, the tinnitus subjects showed greater attention modulation of cortical responses. These differences in attention modulation may serve as a basis for an objective tinnitus biomarker, but this observation first needs further validation in other studies.

Many people with tinnitus reported a subjective worsening of symptoms during the COVID-19 pandemic. Jedrzejczak and colleagues explored the subjective experience of tinnitus during the pandemic, focusing on whether the pandemic-induced stress and isolation affected tinnitus sufferers’ perception of their condition and neural activity (contribution 3). While no difference in THI scores was observed, their qEEG assessment suggests differences in neural activity between groups. It is surprising yet reassuring that the social and emotional impact of the pandemic did not worsen the tinnitus burden. 

The insight that the central auditory pathway plays a major role in the tinnitus mechanism has led to research using different forms of neurostimulation. Non-invasive and invasive central neuromodulation directly impacts central tinnitus pathways, but even peripheral stimulation may lead to central auditory system modulation. Deklerck and colleagues examined the impact of Cochlear Implants (CIs) on tinnitus in 72 patients with severe to profound hearing loss (contribution 4). CIs have demonstrated effectiveness in reducing tinnitus. However, there remains high variability in outcome, including the induction or aggravation of tinnitus in a minority of patients. The authors found no demographic or hearing-related predictors for tinnitus improvement except for tinnitus severity at baseline. Residual inhibition of tinnitus was noted in 80% of patients after the removal of the processor. These findings support CIs’ role in reducing tinnitus severity, potentially through both peripheral masking and central auditory system modulation.

The critical reviews in this Special Issue highlight the challenges faced by neuromodulation trials for tinnitus. The first review by Basner and colleagues focused on an invasive form of neuromodulation, namely Deep Brain Stimulation (DBS) (contribution 5). Basner and colleagues state that early studies and trials on DBS for tinnitus show promising results, targeting areas like the caudate nucleus and medial geniculate body. However, the optimal target for DBS remains unclear due to limited clinical data. Despite the small number of cases, the authors conclude that DBS has demonstrated safety and efficacy and suggest that next steps are needed to establish the optimal DBS targets and refine treatment protocols for tinnitus.

In contrast, Hoare and colleagues conducted a systematic review on non-invasive neuromodulation treatments, focusing on those that are hypothesized to alter cortical oscillatory activity and, thus, reverse or modify pathological synchronous activity linked to tinnitus (contribution 6). The outcomes of interest included tinnitus symptom severity, depression, anxiety, quality of life, oscillatory power, and adverse effects. Acoustic neuromodulation showed limited effectiveness. Other techniques, like transcranial alternating current stimulation (tACS), vagus nerve stimulation (VNS), and bimodal stimulation, showed promise but lacked sufficient studies for synthesis. The most evidence-supported treatment was transcranial direct current stimulation (tDCS), which showed potential benefits, particularly with higher doses. However, the effect on tinnitus does not appear to be sustained.

It is important to realize that variability in tinnitus characteristics (e.g., pitch, loudness, duration) as well as comorbid conditions such as hearing loss, hyperacusis, anxiety, and depression can influence treatment outcomes. Studies should classify and account for these differences.

Altogether, exploration of the central aspects of tinnitus and neuromodulation has seen remarkable progress in recent decades. Advanced electrophysiological and imaging techniques have greatly enhanced our understanding. The original studies presented here involved relatively small groups, which may limit the generalizability of findings. In future research, assessing auditory function and hyperacusis and including potential other confounders, such as anxiety and depression, may provide more robust and generalizable insights into the neural correlates of tinnitus. Future trials on neuromodulation treatment for tinnitus should include core outcome sets that represent outcomes most important to patients and stakeholders. We advocate a mechanism-based approach utilizing translational research programs that combine the expertise of neuroscientists on central mechanisms, the expertise of engineers offering advanced neuromodulative techniques, and the expertise of clinicians to advance the field of neuromodulative treatment for tinnitus.

## References

[1] Jarach C.M., Lugo A., Scala M., van den Brandt P.A., Cederroth C.R., Odone A., Garavello W., Schlee W., Langguth B., Gallus S. (2022). Global Prevalence and Incidence of Tinnitus: A Systematic Review and Meta-analysis. JAMA Neurol..

